# Sublingual immunotherapy with a carbamylated monomeric allergoidin cat-allergic patients suffering from rhinoconjunctivitis and/or allergic asthma. A multicenter, cross-sectional survey

**DOI:** 10.1186/1939-4551-8-S1-A69

**Published:** 2015-04-08

**Authors:** Bettina Hauswald

**Affiliations:** 1 Uniklinikum Dresden, Germany

## Background

In this cross-sectional survey our purpose is to evaluate the clinical outcome and safety of allergoid sublingual immunotherapy in cat-allergic patients suffering from rhinoconjunctivitis and / or asthma.

## Methods

All patients were drawn anonymously from twenty-one practices across Germany. For this survey, a questionnaire was designed to standardize the patient interviews that were performed by the investigators. Patients were prescribed monomeric allergoid sublingual tablets for an initial up-dosing therapy and afterwards for maintenance therapy. Primary endpoints were improvement of symptoms, medications score and safety during therapy with monomeric allergoids.

## Results

In total, 70 patients completed the questionnaire. Of 70 patients, 35.7% were males and 64.3% females. Regarding rhinitis symptoms, almost thirty percent (29.1%) of the patients had become symptom-free during the first year of therapy. This number increased to 52.8% of the remaining patients in the second year, and to 80% of the remaining patients in the third year of therapy. Similar results were valid for conjunctivitis with 35.6%, 55.2% and 72.7% from the first to the third year of therapy. For asthmatic patients, 35.1% had become symptom-free during the first year, 58.3% during the second year, and 70% during the third year of therapy. At baseline, usage of asthmatic medication was 1.33 ± 1..48 (mean ± SD) relative to 0.68 ± 1.23 (mean ± SD) at the time of the questionnaire conduct, with p ≤ 0.05. For anti-allergic medication, baseline medication score was 1.01 ± 1.37 (mean ± SD) relative to 0.45 ± 0.87 (mean ± SD), with p ≤ 0.001. Regarding safety, no events of death, no anaphylactic reactions, no serious adverse events, and no systemic adverse reactions occurred. Only seven local adverse reactions were reported in 7 patients. Besides the major endpoints, secondary endpoints such as patients’ improvement of quality of life, patients’ compliance, patients’ satisfaction, and trust in the therapy were remarkably enhanced.

## Conclusions

Monomeric allergoid sublingual tablets show clinical advantages in a practice environment and under real-life setting conditions. The results of other studies could be reinforced by the results of our study, with significant reduction in medication score, symptom improvement, and a considerable safety outcome. It can be claimed that a sublingual monomeric tablet therapy is safe and well tolerated and significantly reduces rhinitis symptoms in cat-allergic patients.

